# Cloning of NruI and Sbo13I restriction and modification sstems in *E. coli *and amino acid sequence comparison of M.NruI and M.Sbo13I with other amino-methyltransferases

**DOI:** 10.1186/1756-0500-3-139

**Published:** 2010-05-24

**Authors:** Zhenyu Zhu, Chandra Sekhar Pedamallu, Alexey Fomenkov, Jack Benner, Shuang-yong Xu

**Affiliations:** 1 New England Biolabs, Inc., 240 County Road, Ipswich, MA 01938, USA

## Abstract

**Background:**

NruI and Sbo13I are restriction enzyme isoschizomers with the same recognition sequence 5' TCG↓CGA 3' (cleavage as indicated↓). Here we report the cloning of NruI and Sbo13I restriction-modification (R-M) systems in *E. coli*. The NruI restriction endonuclease gene (*nruIR*) was cloned by PCR and inverse PCR using primers designed from the N-terminal amino acid sequence. The NruI methylase gene (*nruIM*) was derived by inverse PCR walking.

**Results:**

The amino acid sequences of NruI endonuclease and methylase are very similar to the Sbo13I R-M system which has been cloned and expressed in *E. coli *by phage selection of a plasmid DNA library. Dot blot analysis using rabbit polyclonal antibodies to N6mA- or N4mC-modified DNA indicated that M.NruI is possibly a N6mA-type amino-methyltransferase that most likely modifies the external A in the 5' TCGCGA 3' sequence. M.Sbo13I, however, is implicated as a probable N4mC-type methylase since plasmid carrying *sbo13IM *gene is not restricted by Mrr endonuclease and Sbo13I digestion is not blocked by Dam methylation of the overlapping site. The amino acid sequence of M.NruI and M.Sbo13I did not show significant sequence similarity to many known amino-methyltransferases in the α, β, and γ groups, except to a few putative methylases in sequenced microbial genomes.

**Conclusions:**

The order of the conserved amino acid motifs (blocks) in M.NruI/M.Sbo13I is similar to the γ. group amino-methyltranferases, but with two distinct features: In motif IV, the sequence is DPPY instead of NPPY; there are two additional conserved motifs, IVa and Xa as extension of motifs IV and X, in this family of enzymes. We propose that M.NruI and M.Sbo13I form a subgroup in the γ group of amino-methyltransferases.

## Background

Among the four types of restriction-modification (R-M) systems discovered from microbial sources based on subunit complexity, ATP/GTP requirement, and methylation-dependency, the Type II restriction endonucleases (REases) are useful tools in cleaving DNA into specific fragments for gene cloning and analysis [1]. Type II restriction endonuclease genes are usually accompanied by companion methylase genes encoding methylases that modify the same target sites to avoid self-destruction of genomic DNA or extra chromosomal DNA [2]. In bacterial warfare, foreign or phage DNAs are unmodified by the host resident methylases and therefore subjected to restriction by the companion endonuclease, while "self" DNA is partially or fully modified and thus resistant to Type II endonucelase attack. Over 3500 R-M systems have been found in nature with approximately 300 unique specificities containing 4-8 bp recognition sequences [3]. There are three major types of base modification in bacteria and archaea: 5mC-methylation of the cytosine pyrimidine ring carbon producing 5-methylcytosine, N4-methylcytosine (N4mC), and N6-methyladenine (N6mA) [4-6]. In 5mC methylases such as M.HhaI, there are ten conserved amino acid motifs (blocks) that are arranged in the order of I to X (circular permutation of motifs IX and X in 5mC methylases has also been observed previously) [7,8]. Among the N4mC and N6mA methylases, there are three major groups of methylases, i.e, α, β, and γ, based on the order of the amino acid motifs involved in S-adenosyl-L-methionine (AdoMet, methyl donor) binding (motifs X-I-II-III), catalytic function (motifs IV-V-VI-VII-VIII), and DNA target recognizing (TRD) [9,2]. In the α group of amino-methyltransferases, the AdoMet binding region precedes the TRD and the catalytic region (X-I-II-III-TRD-IV-V-VI-VII-VIII, Motif IV = DPPY). In the β group, the catalytic region is arranged before the TRD and AdoMet binding region (IV-V-VI-VII-VIII-TRD-X-I-II-III, motif IV = DPPY or SPPY). In the γ group, the conserved motifs are arranged in the order of AdoMet binding region, catalytic region, and TRD (X-I-II-III-IV-V-VI-VII-VIII-TRD, motif IV = NPPY).

The NruI restriction endonuclease (REase or R) and methyltransferase (methylase or M) are enzymes isolated from the bacterium *Nocardia rubra *(Comb D.G., Schildkraut I., Greenough L. unpublished results cited in [3]). The NruI endonuclease binds to the symmetric sequence 5'-TCGCGA-3' in double-stranded DNA (dsDNA) and cleaves the DNA between the G and C in both strands (5'-TCG↓CGA-3'), thus producing DNA fragments with blunt ends. Sbo13I endonuclease is an NruI isoschizomer found in the strain *Shigella boydii *C13 (strain # NCTC 9361) [10]. The goal of this work was cloning of the NruI R-M system in *E. coli*. During the initial cloning attempt, the methylase gene selection strategy [11] was not successful in cloning the *nruIM *gene, probably as a result of poor expression of *nruIM *in *E. coli*. Therefore, we sequenced the NruI endonuclease protein and obtained partial N-terminal amino acid sequence, which was then used to design degenerate primers for inverse PCR amplification of the coding sequence. Additional PCR reactions were carried out to amplify the entire NruI R-M system and the *nruIR *gene was successfully cloned in *E. coli*. The Sbo13I R-M system was cloned in *E. coli *by phage selection from plasmid expression libraries. When the M.NruI and M.Sbo13I amino acid sequences were searched against other known amino-methyltransferases in protein database in BlastP analysis [12], very little sequence similarity was detected, except five putative methylases. We propose that M.NruI and M.Sbo13I should be included in the γ group of amino-methyltransferases with two distinct features: motif IV is DPPY instead of NPPY, and two additional amino acid motifs IVa and Xa are also present, which might be involved in DNA target recognition or catalytic activity.

## Methods

### Strains, plasmid vectors, enzymes, primers, and genomic DNA preparation

*E. coli *strain ER2683 is an MM294 derivative (F'ProA^+^B^+ ^*lacI*^*q *^Δ*lacZM15 *miniTn10 (Kan^R^) *fhuA2 *Δ(*lacI-lacA*)200 *glnV44 *e14^- ^*rfbD1*? *relA1*? *endA1 spoT1*? *thi-1 *Δ(*mcrC*-*mrr*)114::IS10) (strain provided by Lise Raleigh, NEB). Plasmid vectors pUC19, pACYC184, restriction enzymes, modification enzymes, Taq and Vent^® ^DNA polymerases were produced at New England Biolabs, Inc (NEB). Degenerate inverse PCR primers were designed from the N-terminal NruI amino acid sequence (MGFLADXDLSYDEINELLTDN). The following primers were made by the NEB Organic Synthesis Division for inverse PCR amplification of the *nruIR *coding sequence:

Pho RTCNGCYAARAANCCCA (R = A/G; N = A/T/C/G; Y = T/C; Pho = 5'Phosphate)

(P270, mixed primers corresponding to aa MGFLAD)

Pho RTCNGCNAGTAANCCCA (P271, mixed primers corresponding to aa MGFLAD)

Pho TAYGAYGARATHAAYGA (P272, mixed primers corresponding to aa YDEINE).

Two-step gradient inverse PCR was carried out using primer pair P270 + P272 or P271 + P272 (DNA denaturing temperature at 95°C for 30 seconds, annealing temperature gradient at 37°C to 55°C for 30 seconds with 0.5°C increase in every cycle, 72°C extension for 2 min, 4 units of Taq DNA polymerase, 200 to 400 ng of endonuclease-digested and self-ligated genomic DNA template for 35 thermocycles).

Genomic DNA was prepared from 10 g of *Nocardia rubra *cells by a modified procedure based on the references [13,14]. Cell paste was resuspended in 35 ml of 0.1 M Tris-HCl, pH 7, 0.1 M EDTA. Cell lysis was carried out by addition of 25 ml of 2 mg/ml fresh lysozyme in 0.1 M Tris-HCl, 0.1 M EDTA, pH 7.6, with incubation at 37°C for 1 hour. Protease K was added to the cell lysate at 0.1 mg/ml, with incubation at 37°C for 1 hour. To further facilitate cell lysis, SDS and sarcosyl solutions were added to 0.1% and 0.9% final concentration, respectively, with incubation at 55°C for 1 hour. The lysed cells were subjected to Phenol-CHCl_3 _extraction (3 ×), and CHCl_3 _extraction (2×). The nucleic acids were dialyzed in 4 L of TE buffer (10 mM Tris-HCl, pH 7.5, 0.1 mM EDTA) at 4°C overnight (2×). RNA was removed by digestion with RNaseA at 37°C for 1 hour. The quality of genomic DNA was analyzed on a 0.8% agarose gel. The size of the genomic DNA was estimated to be larger than 10 kb.

*E. coli *cell extracts were prepared as described previously [15]. Native NruI endonuclease was purified from *Nocardia rubra *cell lysate by chromatography through following columns: heparin Hyper-D, Source Q, Affigel blue, ceramic HTP and Mono Q columns. N-terminus amino acid sequences were determined by the Edman degradation method [16]. NruI endonuclease activity was carried out in a 1× NruI buffer: 100 mM KCl, 50 mM Tri-HCl, pH 7.7, 10 mM MgCl_2_, in a total volume of 50 μl at 37°C for 1 h on DNA substrate. Plasmid DNA and inverse PCR products were sequenced using the BigDye terminator cycle sequencing kit (Applied Biosystems). Dot blot analysis using N6mA or N4mC antibodies was carried out as described previously [17].

### Bioinformatic analysis

Multiple sequence alignment of M.NruI and M. Sbo13I, and methylase homologs were performed using the PROMALS3D web server [18]. The amino acid sequences of NruI and Sbo13I and putative isoschizomers were analyzed using the Clustal W web server [19]. MAFFT (v 6.0), using the minimum linkage method, was used to calculate distances from the PROMALS3D alignment and an unrooted distance tree was constructed for the M.NruI/M.Sbo13I family of N6mA methylases [20]. Cluster analysis of multiple sequences was carried out using CLANS to visualize the clustering (formation of closely related protein families) among the M.NruI/M.Sbo13I group and known N6mA methyltransferases. CLANS is a Java utility tool based on the Fruchterman-Reingold graph layout algorithm [21].

## Results and Discussion

### Attempt to clone NruI endonuclease gene (*nruIR*) and methylase gene (*nruIM*) by methylase selection method

At first, the methylase selection method [11,13] was used to clone the *nruIM *gene, but without success. It's known that M.NruI exists in the native strain because the genomic DNA from *Nocardia rubra *is resistant to NruI digestion (data not shown). However, the cloning of the NruI methylase gene (*nruIM*) proved to be extremely difficult. Dozens of genomic DNA libraries were constructed with different vectors and various enzyme digested genomic DNA fragments. The plasmid DNA libraries were subjected to NruI digestion and the digested DNA was then transferred into a McrBC^- ^Mrr^- ^*E. coli *host. But no true resistant clones were ever recovered (data not shown). The negative results may be due to the low expression of the methylase gene in *E. coli *and poor modification of NruI sites on the plasmid. It's known that *Nocardia *has a very high GC contents (the GC contents of *Nocardia farcinica *IFM10152 are 70%) [22], and genes from *Nocardia *may not be expressed efficiently in *E. coli*.

### Purification of native NruI and N-terminal amino acid sequencing

Native NruI was purified from *Nocardia rubra *cell lysates by chromatography through the following columns: heparin Hyper-D, Source Q, Affigel blue, ceramic HTP and Mono Q columns. The starting crude extracts had 5.6 × 10^6 ^units, and the final purified enzyme contained 1.5 × 10^6 ^units. The enzyme preparation was only functionally pure, free of DNA, RNA and other nonspecific nucleases (data not shown). The purified NruI endonuclease was subjected to SDS-PAGE and more than ten protein bands were identified (data not shown). The four bands between 25 kDa and 47.5 kDa were selected and their N-terminus amino acid sequences were determined by Edman degradation method (most Type IIP restriction enzymes are in the range of 20 to 50 kDa in molecular masses, see REBASE). Only the protein band close to 25 kDa produced a protein sequence: MGFLADXDLSYDEINELLTDN (X, unidentified amino acid residue). The rest of the protein bands did not yield meaningful data (data not shown).

### Design of degenerate inverse PCR primers and amplification of *nruIR *gene by inverse PCR

Genomic DNA was prepared from *Nocardia rubra *by phenol-chloroform extractions. One μg of genomic DNA was digested with individual restriction enzymes with 4-6 recognition sequences. Digested DNA was then purified and self-ligated at a low DNA concentration. The ligated DNA was further purified by Qiagen spin columns. The final volume was 50 μl and 10 μl was used as the template for degenerate inverse PCR.

**Figure 1:**
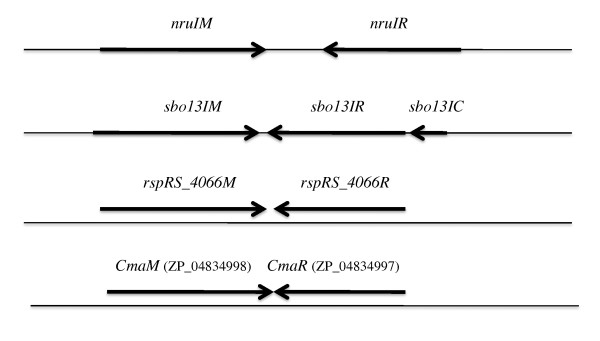
**Gene organization of the NruI and Sbo13I R-M systems**. The *nruIM *and *nruIR *genes are transcribed in the opposite direction. There is a gap of 350 bp non-coding sequence between the two genes. The controller gene *sbo13IC *and *sbo13IR *gene are transcribed in the same direction. The *sbo13IM *gene is in the opposite orientation. The NruI and Sbo13I R-M sequences have been submitted to GenBank and assigned the accession number: HM022156 and HM236832, respectively.

**Figure 2:**
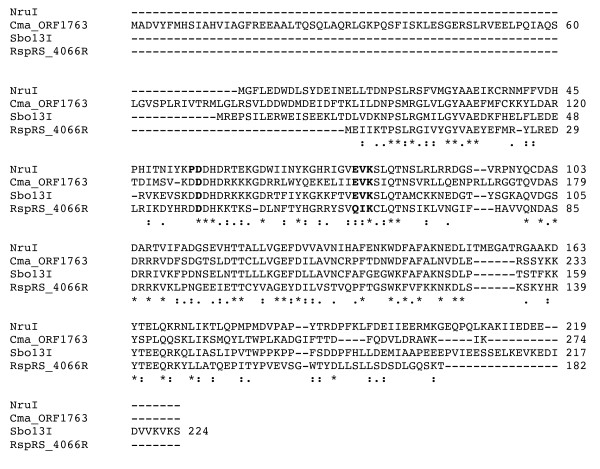
**CLUSTAL W multiple sequence alignment of NruI, Sbo13I, and two putative restriction enzymes CmaR (protein ID: ZP_04834997, a helix-turn-helix domain protein found in *Corynebacterium matruchotii*, ATCC 14266) and RspRS_ORF4066 found in the sequenced genome of *Roseiflexus *sp. RS-1**. The marks "*" and ":" indicate identical aa residues and aa residues with similar properties respectively.

The primer pair P271 and P272 with different templates produced multiple PCR products. A total of 26 PCR DNA fragments were purified from low-melting agarose gels and sequenced by primers P271 and P272. DNA sequencing of four inverse PCR products generated a 774-bp sequence contig. When the DNA sequence was translated into amino acid sequence and compared to known REases in REBASE, it shows 55% similarity and 46% identity to Sbo13I endonuclease, an NruI isoschizomer (Figures 1 and 2, see below). It is known that isoschizomer REases sometimes share ~30% to 95% amino acid sequence identity [3,23]. So this high degree of sequence similarity confidently identified this 774-bp contig to be the gene fragment encoding part of the NruI REase. The other inverse PCR products were presumably amplified from genomic DNA non-specifically and discarded.

The remaining DNA of the NruI R-M system was obtained by further inverse PCRs. The NruI R-M system consists of R and M genes running in the opposite direction and the two genes were separated by a large gap (326-bp non-coding sequence) (Figure 1). The *nruIM *gene is 804 bp and encodes a polypeptide of 267 amino acids, and the *nruIR *gene is 660 bp coding for a polypeptide of 219 amino acids (Figure 1). The N-terminus of the translated *nruIR *is MGFLEDWDLSYDEINELTDN (Figure 2), which closely matches the N-terminal aa sequence derived from the native NruI endonuclease (MGFLADXDLSYDEINELLTDN). Another new finding was that when the amino acid sequence of M.NruI was compared to other known amino-methyltransferases in GenBank and REBASE by blastx/blastp, M.NruI only shows sequence similarity to M.Sbo13I and a few other putative methylases. M.NruI/M.Sbo13I and a few others share a unique amino acid sequence similarity among the DNA amino-methyltransferases. This high degree of amino acid sequence similarity was not found in other N4C or N6A methylases (more detailed sequence comparison below).

BlastP analysis using NruI as a query also identified two additional putative endonucleases Cma ORF1763 (GenBank ID: ZP_04834997) from *Corynebacterium matruchotii *ATCC 14266 (annotated as a helix-turn-helix XRE-family DNA binding protein) and RspRS ORF4066 found in the sequenced genome of *Roseiflexus *sp. RS-1 (GenBank accession number CP000686, coordinate 5095066-5095614, complement strand sequence). Figure 2 shows the Clustal W multiple sequence alignment of NruI, Sbo13I, Cma ORF1763 (ZP_04834997), and RspRS ORF4066R. The predicted catalytic sites in NruI, Sbo13I and Cma ORF1763 (ZP_04834997) are PD-X_21_-EVK or DD-X_21_-EVK. In RspRS ORF4066R, however, the predicted catalytic residues are DD-X_20_-QIK (Mrr endonuclease-like catalytic site) [24]. The endonuclease activity of Cma ORF1763 (ZP_04834997) and RspRS ORF4066R remains to be examined by experimentation. There are putative companion methylases (ZP_04834998; YP_001278360 = RspRS_ORF4066M, complete genome coordinate 5094219 to 5095064) adjacent to Cma ORF1763 (ZP_04834997) and RspRS ORF4066R respectively that show significant sequence similarity to M.NruI/M.Sbo13I (see below). The gene organization of the two putative NruI isoschizomers is shown in Figure 1. Similar to NruI and Sbo13I, the homologous R-M genes are transcribed in opposite directions, but lacking a large non-coding region between the R-M genes.

### Cloning of Sbo13I Restriction modification system in *E. coli*

Sbo13I restriction endonuclease and modification methylase were derived from *Shigella boydii *C13 [10]. Sbo13I and NruI recognize the same DNA sequence TCG/CGA and cleave at the same position. Previous results indicated that the Sbo13I R-M system was encoded by a resident plasmid (data not shown). Plasmid DNA of *Shigella boydii *C13 was prepared by the cesium chloride-ethidium bromide equilibrium density centrifugation method. One isolate (A5) was used to map the 5.4 kb restriction system-containing plasmid for single restriction sites. The purified A5 isolate DNA was then digested with ClaI, HindIII and NsiI, respectively and ligated into pUC19 or pBR322 (CIP-treated with compatible ends). The ligated DNA was used to transform *E. coli *strain RR1 and selected on LB agar Amp plates. Amp^R ^transformants were picked into 200 μl of LB with antibiotic into micro-titer plates. Master plates were prepared by stamping on LB with antibiotic. Replica plates were prepared by stamping onto four levels of T7 phage: 10^9^, 10^7^, 10^5^, and 10^3 ^phage/plate. Individual colonies which survived at all levels of phage infection were considered to be phage-resistant and hence likely to carry an active restriction system.

A number of plasmids were found to carry ClaI or NsiI fragments of approximately 5.4 kb in length and these plasmids were resistant to Sbo13I digestion. These plasmids were subsequently shown to carry the complete Sbo13I R-M system. The recombinant plasmid pBLSboC13M8.1 which carries the gene encoding the Sbo13I restriction endonuclease and methylase was transferred into *E. coli *strain RR1 by transformation. Approximately, 10^5 ^units of Sbo13I REase per gram of wet cell paste were produced from the expression strain (data not shown). The *sbo13IR *and *sbo13IM *genes were sequenced by the Sanger sequencing method (data not shown). The gene organization of the Sbo13I R-M system is shown in Figure 1. Similar to the NruI R-M system, the *sbo13IR *and *sbo13IM *genes are transcribed in opposite directions. In addition, there is a controller gene *sbo13IC *in front of the *sbo13IR *gene, which may regulate the transcription of the R gene, by analogy to the PvuII and BclI controller proteins [25,26].

The NruI endonuclease was expressed in pre-modified *E. coli *hosts and the recombinant NruI endonuclease was purified (ZZ, SYX, unpublished results).

### Dot blot analysis to determine N6mA modification for M.NruI and M.Sbo13I

**Figure 3:**
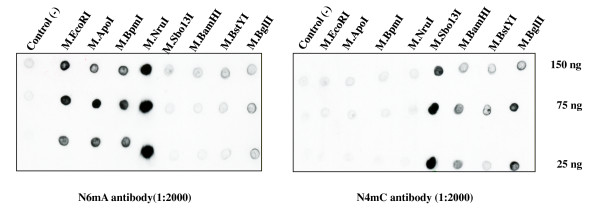
**Dot blot analysis of M.NruI and M.Sbo13I modified genomic DNA using rabbit antibodies against N6mA-methylated DNA (left panel) or N4mC-methylated DNA (right panel)**. M.EcoRI and M.ApoI are known N6mA methylases; while M.BamHI, M.BglIII and possibly M.BstYI are N4mC methylases. Negative control total DNAs were prepared from transformants of empty vectors. Three DNA dilutions (150 ng, 75 ng, and 25 ng) were spotted on the membrane for detection.

The *nruIM *gene with consensus ribosome binding site and spacer was amplified by PCR and cloned into pACYC184. The plasmid DNAs pACYC-*nruIM *and pUC19-*sbo13IRM *were transferred into *E. coli *DB24 (deficient in Dam methylase, Dcm methylase, McrBC, and Mrr) and total DNA were isolated. Methylation activity was measured using a dot blot assay [17] employing primary rabbit polyclonal antibodies specific for N6-methyladenine (N6mA) or N4-methylcytosine (N4mC). M.NruI-modified DNA shows positive signal at 25 ng to 150 ng DNA dot blot, similar to the signal generated by the positive control N6 adenine methylases M.EcoRI, M.ApoI, and M.BpmI (Figure 3, left panel). M.Sbo13I-modified total DNA generated background signal at 25 ng to 150 ng with the same antibodies (Figure 3, left panel, compare negative control-empty vector with M.Sbo13I-modified DNA). Dot blots using anti-N4mC antibodies produced a strong signal from M.Sbo13I-modified total DNA, similar to the signals generated by two known N4mC methylases M.BamHI or M.BglII-modified DNA (Figure 3, right panel) [27,28]. Based on the dot blot analysis we concluded that M.NruI is probably an N6-adenine methylase that most likely modifies the external A in TCGCGA sequence. Consistent with this result, Dam methylation at the overlapping TCGCGAtc sequence also blocks NruI digestion [29]. M.Sbo13I is probably an N4mC methyltransferase based on the following evidences: 1) rabbit N4mC antibodies generated strong dot blot signal; 2) plasmid carrying *sbo13IM *gene was not restricted by Mrr endonuclease in transformation experiment (data not shown); 3) Dam methylase modified DNA sequence TCGCG^m^Atc can be cleaved by Sbo13I [29]; 4) M.Sbo13I is somewhat similar to a known N4mC methyltransferase M.Hpy99I although the similarity is at the border line. The final proof of modified bases by M.Sbo13I may be derived from DNA oligos modified by purified M.Sbo13I, which remains to be studied.

### Sequence comparison of M.NruI and M. Sbo13I

Comparison of M.NruI and M.Sbo13I amino acid sequences indicates that they are significantly similar to each other (49% aa sequence identity). Furthermore, these enzymes may form a new family (cluster) of methyltransferases based on their lack of similarity to many other known methyltransferases available in REBASE. Alternatively, they may belong to a subgroup of already established α, β and γ groups of N6mA methyltransferases (Malone et al. 1995).

The other members of this family (cluster) were identified by querying M.NruI and M.Sbo13I protein sequences against non-redundant protein sequences (NR) and REBASE databases using BlastP searches. Significant hits with an expectation value <1E-20 and sequence length of more than 200 amino acids were considered potential M.NruI and M.Sbo13I homologs (putative isoschizomer). M.NruI and M.Sbo13I enzymes share amino acid sequence similarities with proteins YP_001278360 (RspRS_4066 from *Roseiflexus *sp. RS-1), ZP_01906951 (PPSIR1_36012 from *Plesiocystis pacifica *SIR-1), ZP_03711821 (CORMATOL_02672 from *Corynebacterium matruchotii*), YP_001432817 (Rcas_2728 from *Roseiflexus castenholzii *DSM 13941). The GenBank protein and gene IDs are described as following: ZP_01906951 (gi_149918461, PPSIR1_36012 from *Plesiocystis pacifica *SIR-1), ZP_03711821 (gi_225022629, CORMATOL_02672 from *Corynebacterium matruchotii*), ZP_04834998 (gi_252123721, M.Cma ORF1763, ATCC_14266 from *Corynebacterium matruchotii*), and M.Rca13941ORF2728P (DSM 13941 from *Roseiflexus castenholzii*).

**Figure 4:**
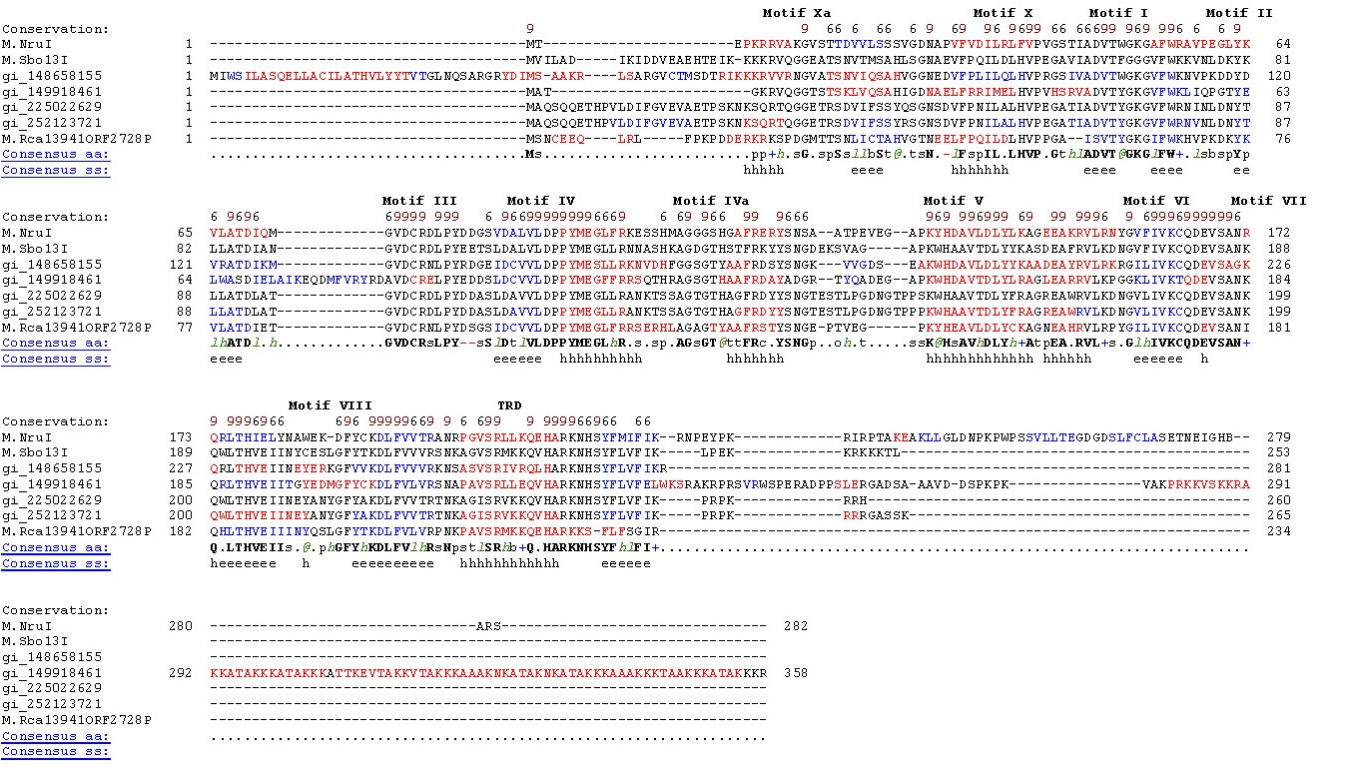
**Conserved amino acid sequence blocks in the multiple sequence alignment of M.NruI and M.Sbo13I family sequences**. The homologous sequences found in GenBank are: gi_148658155 (YP_001278360), gi_149918461 (ZP_01906951), gi_225022629 (ZP_03711821), gi_252123721 (ZP_04834998) and putative methyltransferase M.Rca13941_ORF2728P. The consensus amino acid residues are indicated below the 7 actual sequences. The h and e indicate secondary structural prediction for α-helix (indicated in red) and β-strand (indicated in blue) respectively. Conserved amino acid motifs (blocks) I through X are shown above the amino acid sequences.

To identify the conserved amino acid motifs (blocks) we performed a multiple sequence alignment of M.NruI/M.Sbo13I family enzymes using PROMALS3D. Figure 4 illustrates the conserved amino acid sequence motifs (blocks) in the alignment of the M.NruI/M.Sbo13I family sequences. Motifs are labelled using the nomenclature of Posfai et al. and Malone et al. [7,9]. The putative motifs identified in the M.NruI/M.Sbo13I family have some similarities to standard motifs defined in the reference [9] which are listed as below.

i.	Motif I contains a core G-loop (Gly-X-Gly) tripeptide that may bind the methionine moiety of AdoMet. X indicates any amino acid.

ii.	Motif II contains a charged residue at the end of a predicted β strand which may interact with ribose hydroxyls of AdoMet. It is followed by a bulky hydrophobic side-chain (Ile or Leu) that may make van der Waals contact with the adenine of AdoMet.

iii.	Motif IV contains a P-loop, DPPY motif, which may form the active site, along with motifs V to VIII. Superficially, the DPPY motif suggests that M.NruI and M.Sbo13I may be related to the α group of N6mA methyltransferases. However, the phylogenetic distance analysis indicates otherwise.

iv.	Motif V contains the consensus Asp-Leu-Tyr-X-X-Ala-(Gly/Ser) which differs from the standard motif V (Asn/Asp-Leu-Tyr-X-X-Phe-(Leu/Val/Ile) defined in the literature for the γ group of N6mA methyltransferases. The first three amino acids are the same as standard amino acids, but the last two amino acids are different.

v.	Motif VI starts with Gly and ends with a hydrophobic amino acid (Val) which is very similar to the standard motif defined in literature.

vi.	Motif VII is highly hydrophobic which is consistent with the standard motif. This hydrophobic region may interact with the target DNA adenine.

vii.	Motif X is located at the N-terminus of the primary sequence and contains conserved hydrophobic residues.

**Figure 5:**
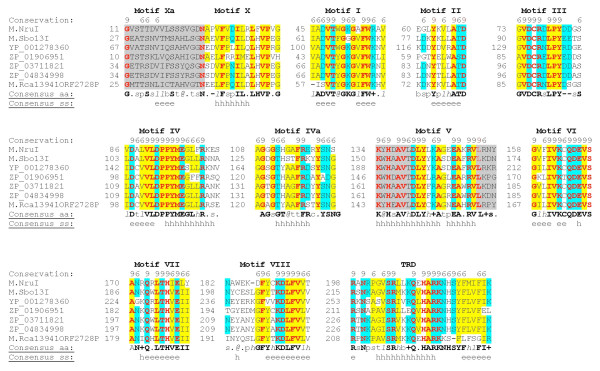
**Conserved amino acid (aa) sequence motifs (blocks) in the alignment of M.NruI/M.Sbo13I family**. Red bold letter indicates invariant aa and h and e indicate secondary structural element α-helix and β-strand respectively. Shaded regions indicate extensions of respective motifs. The number to the left of each sequence indicates the absolute location of the first residue of the segment within the whole sequence. Shaded yellow background indicates hydrophobic aa and shaded blue aa indicates polar or charged residue. Conserved amino acids are grouped as (E, D, Q, N), (V, L, I, M), (F, Y, W), (G, P, A), (K, R) and (S, T), using standard one-letter abbreviations (Malone et al. 1995).

**Figure 6:**
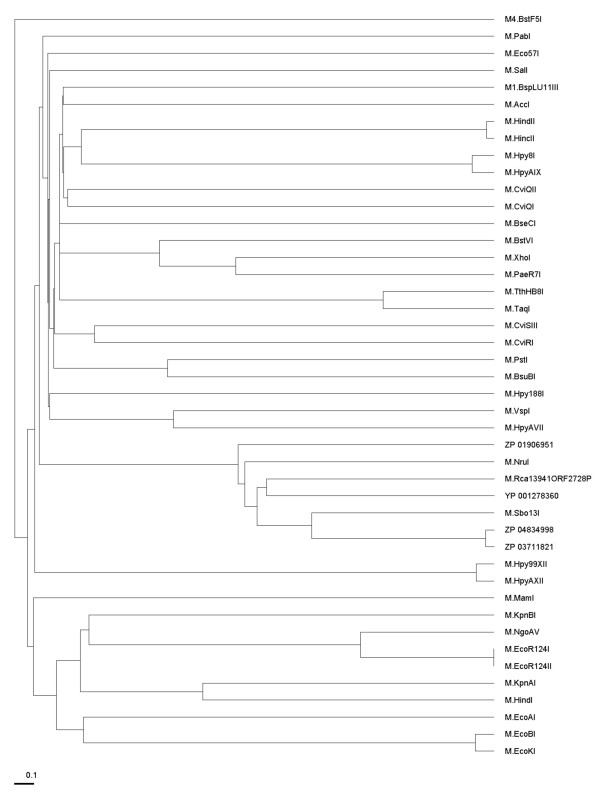
**An unrooted phylogenetic tree of M.NruI and M.Sbo13I in relationship with other DNA N6mA methyltranferases in the γ group**. MAFFT (version 6) with minimum linkage method was used to calculate distances from PROMALS3D multiple sequence alignment.

**Figure 7:**
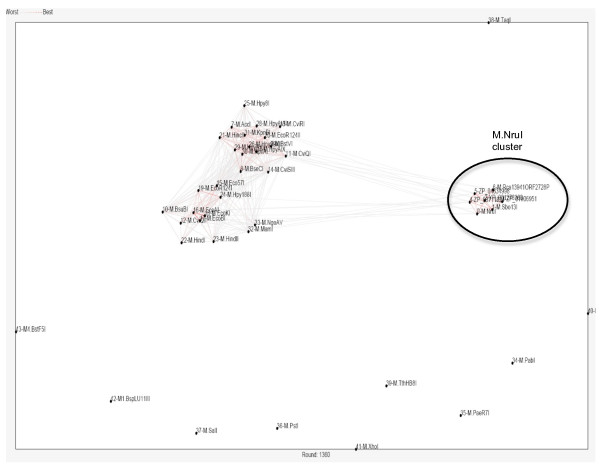
**Cluster analysis of γ group N6mA methylases**. Clustering of multiple sequences was carried out using CLANS to visualize the formation of protein family among the M.NruI/M.Sbo13I group and other γ group known N6mA methyltransferases.

Figures 4 and 5 also show that Motifs X and V have some extra conserved regions compared with the standard motifs. We also identified two additional amino acid motifs (blocks) that may be involved in DNA target site recognition or methyl donor cofactor binding. Motif IVa is located between putative motif IV and V, and Xa is located at the N-terminus (motifs IVa and Xa could be extension of motifs IV and X). To identify to which clade (group) of methyltransferases M.NruI/M.Sbo13I belongs, we performed a phylogenetic analysis of M.NruI/M.Sbo13I to known groups of N6-methyladenine methyltransferases. To construct the phylogenetic tree, a multiple sequence alignment of M.NruI/M. Sbo13I and homologs was obtained using the PROMALS3D web server [18] and the three recognized groups of Type II N6mA methyltransferases (α, β and γ) separately. MAFFT (version 6.0) using the minimum linkage method was used to calculate distances from the PROMALS3D alignment and an unrooted phylogenetic tree was constructed for the M.NruI/M.Sbo13I and closely related enzymes and other Type II N6mA methyltransferases (Figure 6). From the unrooted distance tree, it is apparent that M.NruI/M.Sbo13I and their relatives form a unique clade (subgroup) among the γ group N6mA methyltransferases. Furthermore, we performed cluster analysis of sequences using CLANS to visualize the similarities within the M.NruI/M.Sbo13I family and other known groups of N6mA methyltransferases. CLANS is a Java utility tool based on the Fruchterman-Reingold graph layout algorithm [21]. Figure 7 clearly illustrates that M.NruI/M.Sbo13I and related enzymes forms their separate cluster that is distinct from other known γ group N6mA methyltransferases. It is very likely that although the protein structure and function of the M.NruI/M.Sbo13I family is conserved among members of N6mA methyltransferases, their primary amino acid sequences have diverged quite significantly from the common ancestor. This conclusion was supported by the BlastP results that M.NruI amino acid sequence query failed to identify any significant hits to known N6mA methylases in GenBank. BlastP using M.Sbo13I as query, however, found some weak amino acid sequence homology with M.Hp99I and M.NgoMXV, which are known N4mC methylases.

**Figure 8:**
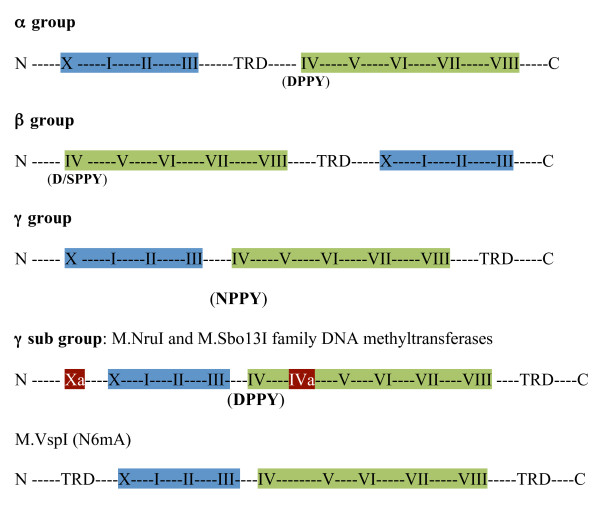
**Conserved amino acid sequence motif (block) organization in different groups of amino DNA methyltransferases**. Amino acid motifs in shaded blue (motifs X, I, II, III) contain the AdoMet-binding region, and the motifs in shaded green harbour the catalytic region (motifs IV - VIII). The extra motifs (blocks) Xa and IVa in the M.NruI/M.Sbo13I sub-group are also shown. TRD, DNA target-recognizing domain. N, N-terminus; C, C-terminus.

Figure 8 further illustrates the motif organization of established groups of methyltransferases and the M.NruI/M.Sbo13I family. M.VspI [30] appears to share the motifs organization of the M.NruI/M.Sbi13I family, but belongs to a different clade in the unrooted phylogenetic tree.

## Conclusions

Both NruI and Sbo13I R-M systems have been cloned in *E. coli*. BlastP analysis of proteins in GenBank indicated that two ORFs are probable isoschizomers of NruI/SboI or enzymes with similar recognition sequence. BlastP analysis of proteins in GenBank using the M.NruI amino acid sequence as a query revealed five additional putative amino-methylases that share similar motif organization. The M.NruI/M.Sbo13I family enzymes appear to belong to the γ group of amino-methyltransferases with two distinct features: Motif IV contains DPPY instead of NPPY sequence; two additional motifs (IVa and Xa) are also present, which may be involved in target recognition and AdoMet binding. Despite the extensive amino acid sequence similarity between M.NruI and M.Sbo13I, M.NruI is most likely an N6mA amino-methyltransferase and M.Sbo13I is likely an N4mC methyltransferase.

## Competing interests

The authors have a competing financial interest in NruI endonuclease. They have no financial interests in Sbo13I endonuclease, M.NruI, and M.Sbo13I.

## Authors' contributions

ZZ cloned and expressed NruI R-M system, CSP performed bioinformatic analysis, AF performed dot blot analysis, JB cloned Sbo13I R-M system, SYX performed data analysis, CSP and SYX wrote the manuscript.
